# The origin of modern North Africans as depicted by a massive survey of mitogenomes

**DOI:** 10.1038/s41598-025-12209-x

**Published:** 2025-07-25

**Authors:** Giulia Colombo, Elisabetta Moroni, Alessandro Raveane, Nicola Rambaldi Migliore, Vincenzo Agostini, Rosalinda Di Gerlando, Claudio Fiorini, Leonardo Caporali, Francesca Gandini, Elena Raimondi, Eugenia D’Atanasio, Hovirag Lancioni, Valerio Carelli, Maria Pala, Beniamino Trombetta, Andrea Novelletto, Jean-Michel Dugoujon, Alessandro Achilli, Antonio Torroni, Martin B. Richards, Fulvio Cruciani, Ornella Semino, Anna Olivieri

**Affiliations:** 1 Department of Biology and Biotechnology “Lazzaro Spallanzani”, University of Pavia, Pavia, 27100 Italy; 2 Molecular Biology and Transcriptomic Unit, IRCCS Mondino Foundation, Pavia, Italy; 3 IRCCS Istituto delle Scienze Neurologiche di Bologna, Programma di Neurogenetica, Bologna, 40139 Italy; 4 B-Cell Neoplasia Unit and Strategic Research Program on CLL, IRCCS Ospedale San Raffaele, Milan, Italy; 5 Medical School, Università Vita-Salute San Raffaele, Milan, Italy; 6 Istituto di Biologia e Patologia Molecolari (IBPM), Consiglio Nazionale delle Ricerche (CNR), Rome, 00185 Italy; 7 Department of Chemistry, Biology and Biotechnology, University of Perugia, Perugia, 06123 Italy; 8 Department of Biomedical and Neuromotor Sciences, University of Bologna, Bologna, 40139 Italy; 9 School of Applied Sciences, University of Huddersfield, HD1 3DH, Queensgate, Huddersfield, England UK; 10 Dipartimento di Biologia e Biotecnologie “Charles Darwin”, Sapienza Università di Roma, Rome, 00185 Italy; 11 Department of Biology, University of Rome “Tor Vergata”, Rome, 00133 Italy; 12 Laboratoire d’Anthropologie Moléculaire et Imagerie de Synthèse (AMIS), UMR 5288, Université Paul Sabatier Toulouse III, Toulouse, 31073 France

**Keywords:** Population genetics, Biological anthropology

## Abstract

North Africa occupies a unique geographical position in the Mediterranean basin. The genetic profile of modern North African populations is the result of ancient and recent movements of people and periods of isolation. In this paper, we provide a comprehensive mitogenome analysis based on unbiased population sampling from across North Africa including a dataset of 733 modern (238 of which new) and 43 ancient mitogenomes from Morocco, Algeria, Tunisia, Libya and Egypt, compared with modern mitogenomes from Eurasia and sub-Saharan Africa. Our analyses, based on a phylogeographic and demographic approach suggest that mitogenome variability in North African populations (i) can be traced back to three main origins - Eurasia, North Africa and sub-Saharan Africa - and (ii) has been defined during crucial key time periods - from the Palaeolithic to the last millennia. In particular, we identified a signal for population movements from sub-Saharan Africa to North Africa during the favourable climatic period of the Green Sahara, providing evidence from complete mitogenomes that the human dispersals associated with this period also involved females.

**Supplementary Information:**

The online version contains supplementary material available at 10.1038/s41598-025-12209-x.

## Introduction

The genetic profile of modern North African populations is the result of both long periods of isolation and the variegated contribution of people who arrived in the region from different geographic sources at different times for more than 40,000 years.

The first securely dated evidence of the early phase of *H. sapiens* evolution in Africa is represented by the fossils discovered at the Jebel Irhoud site, in present-day Morocco dated to around 315 ± 34 thousand years ago (kya)^1^. Several successive archaeological cultures have also appeared before the Neolithic (such as the Middle Palaeolithic Aterian, the Upper Palaeolithic Dabban in Cyrenaica and Iberomaurusian, and the Mesolithic Capsian or Oranian) across North Africa^2–4^. The Neolithic and subsequent periods saw the rise and spread of populations of the Maghreb, known as Berbers, Amazigh or Imazighen. These names refer to diverse cultures characterised by the same linguistic group, the Berber languages, which belong to the Afro-Asiatic family. Neolithic movements also involved the arrival of populations from both Europe and the Middle East^5,6^while historical times are associated with the advent of several populations from the Mediterranean basin, including Phoenicians (from ~ 1200 B.C.), Romans (from the IInd century B.C.), Vandals, and Byzantines (from ~ 400 A.D.). The arrival of the Arabs in the Maghrebi regions (in north-west Africa) from the VIIth century A.D. had had the greatest impact on Berber cultures, bringing the consequent spread of the Arab language and Arabisation of Berber populations. Finally, in colonial times, the region saw the arrival of Europeans and Ottoman Turks^7^.

During the 1990s, analyses on classical markers^8^ indicated that a major role in shaping the genetic landscape of North Africa had been played by its geographic position. The region is delimited by the Mediterranean to the north, the Atlantic in the west, the Nile in the east, and the Sahara Desert to the south. At different times these served as either point of access or physical barriers, with two consistently accessible waypoints to the outer regions represented by the Sinai towards the Levant, and the Strait of Gibraltar towards Europe. This likely contributed to the establishment of a broadly east-west gradient of genetic diversity^9^.

In addition, the isolation of North Africa from sub-Saharan Africa has varied over the millennia, due to strong periodic climatic fluctuations that affected the Sahara, the world’s largest desert, alternating arid and humid periods^10,11^. The most recent African Humid Period, the so-called last Green Sahara that peaked 9 to 6 kya^12^made possible settlements and movements of human groups in and throughout the Saharan belt^13^. After 5 kya, rapid desertification established a geographic barrier, limiting human movements across this vast region until the domestication of the dromedary and consequent expansion of trans-Saharan trade, likely around the end of the second millennium BC^14^. This relative isolation of northern regions perpetuated the profound genetic difference from the rest of the African continent established during the Late Palaeolithic, so that North Africa is genomically much closer to West Eurasian than sub-Saharan populations^15,16^.

However, despite the paucity of genetic data from North African populations in comparison with the rest of the African continent and Eurasian populations^17^recent genomic and archaeogenomic data indicate that the picture is more complex than previously thought^15–21^. Modern genomics points to four major genetic ancestries to explain the genetic variability of North African populations with three of them, the sub-Saharan, Middle Eastern, and European components, representing genetic inputs from surrounding populations that at different times left their traces in North Africa, and the fourth one, also known as Maghrebi, typical of North Africa and ancestral^22^. This component is also supported by the mitochondrial DNA (mtDNA) haplogroups U6 and M1, the only two out-of-Africa haplogroups primarily introduced in the region^23–25^ and is the legacy of an early “back-to-Africa” dispersal event from western Eurasia that dates back to pre-Holocene times.

This picture has recently been refined using ancient genomics, although North African climatic conditions render ancient DNA (aDNA) preservation particularly poor. The most ancient remains analysed so far are from the archaeological site of Taforalt in Morocco, dating to 13.9–15.1 kya^18^. They belong to the Iberomaurusian culture, which spanned the Last Glacial Maximum and the start of the Holocene (roughly equivalent to Epipalaeolithic times in the Near East) and are characterised entirely by the genomic Maghrebi component mentioned above U6 and M1 lineages. U6 is also seen at the only other pre-Neolithic sites analysed in Morocco and, more recently, Algeria and Tunisia^26^. Interestingly, when additional early and late Neolithic sites in Morocco were assessed, it emerged that early Neolithic North Africans were characterised by both Maghrebi and sub-Saharan genomic components, while late Neolithic individuals were mainly influenced by a Mediterranean-like ancestry, probably brought by the Neolithic expansion that occurred first from the Iberian Peninsula through the Gibraltar straits and later from the Near East^15,26^. Indeed, genome-wide sub-Saharan ancestry fell from around a quarter in the Palaeolithic and Early Neolithic to less than 10% by the Late Neolithic according to these datasets, although the Guanches, thought to have settled the Canary Islands from Morocco ~ 2.5 kya, retained about 15% sub-Saharan ancestry^15^ (Fig. 2 and supplementary notes 6 and 7; cf. also^27^ Fig.S3 corrected).

More recent individuals from the sites of Kerkouane in North Tunisia^20^ and Kulubnarti in Nubia^19^dating back to the mid-Iron Age and late Iron Age, respectively, revealed that the genetic history of North African regions was strongly influenced by the increased rate of mobility across the Mediterranean, bringing to share ancestries within various populations of the entire basin.

The maternally inherited mtDNA has typically been used for one or other of two different kinds of analyses: (i) molecular dissection and characterisation of selected mtDNA lineages/haplogroups; (ii) population survey of unselected samples. Modern North African population analyses have been mostly limited to the mtDNA control-region^28–31^whereas complete mitogenomes can provide a much more detailed and precise picture^16,32–40^. To date, only one study surveyed a population-based dataset^40^but this was restricted to a single Berber population from north-east Algeria. Thus, a comprehensive mitogenome analysis based on unbiased population sampling from across North Africa is still a priority.

To this end, we obtained a dataset of 238 novel complete mitogenomes from modern North Africans, encompassing samples from Morocco, Algeria, Tunisia, and Libya, together with 135 new mitogenomes from the neighbouring countries of Burkina Faso, Niger, Nigeria, Cameroon, and Chad. The latter mitogenomes include samples from two nomadic populations, the Foulbe (from Cameroon, Niger and Nigeria) and the Berber-speaking Tuareg (from Niger, Supplementary Table 1). This new dataset was analysed in the context of 495 modern and 43 ancient previously published mitogenomes from the same geographic regions (Table 1).

**Table 1:** Mitogenomes analysed in the present study.

Macroarea	Country	This study	Literature*	
		Modern (*n* = 373)	Modern (*n* = 495)	Ancient (*n* = 43)
North Africa	Morocco	95	7	26
	Algeria	49	302	–
	Tunisia	34	49	13
	Libya	60	2	2
	Egypt	–	135	2
Total North Africa		238	495	43
Sub-Saharan Africa	Burkina Faso	22	–	–
	Niger	10	–	–
	Nigeria	9	–	–
	Cameroon	29	–	–
	Chad	65	–	–
Total sub-Sahara		135	–	–

*Population surveys only.

## Materials and methods

### Sample

To survey mitogenome sequence variation across North African populations and in surrounding regions, DNA was obtained from 238 North African individuals sampled from four countries, i.e. Morocco, Algeria, Tunisia, and Libya, and 135 from neighbouring countries (Burkina Faso, Cameroon, Chad, Niger and Nigeria), for a total of 373 novel mitogenomes. Specifically, as for North Africa, we sequenced the mitogenomes of 95 Moroccans (35 of Arab origin and 60 from the general population), 49 Algerians, 34 Tunisians, and 60 Libyans, from the general population. The new mitogenomes from neighbouring countries are from 22 individuals from Burkina Faso’s general population, 29 Foulbe from Cameroon, 65 individuals from Chad (including one Goulaye, two Madjingay, two Ngambai, and the remaining 60 from the general population), three Foulbe and seven Tuareg from Niger, and nine Foulbe from Nigeria.

All the DNA samples analysed here were selected from our lab collections, assembled on a voluntary basis over the past decades after obtaining appropriate informed consent and according to all the regulations in effect at that time. The use of the “historical” collections in genomic studies was approved by the Ethical Committee Fondazione IRCCS Policlinico San Matteo (protocol number 0028298/22). DNA was extracted by a standard phenol-chloroform method from peripheral blood after a red blood cell lysing step.

### Sequencing of entire mitogenomes and analysis of the sequences obtained

The complete sequences of the 373 North African and sub-Saharan African mtDNAs were obtained by using next-generation sequencing (NGS) with an Illumina MiSeq at the Molecular Biology and Transcriptomic Unit of the IRCCS Mondino Foundation in Pavia (Italy) and at the IRCCS Institute of Neurological Sciences in Bologna (Italy). The workflow included the amplification of the entire mtDNA within two overlapping long range PCR fragments, PCR purification, Illumina NGS library preparation, pooling and sequencing reactions, following the protocols previously described in Brandini et al.^41^. The files obtained from the MiSeq system were demultiplexed using the software bcl2fastq. FASTQ files were aligned to the reference sequence (rCRS; NC_12920.1)^42^ using the software BWA^43^the obtained BAM files were then filtered and sorted using SAMtools^44^. Sequence variants (nucleotide substitutions and indels) were then called using HaplotypeCaller, a tool within the Genome Analysis Toolkit (GATK)^45^. BCFtools^46^ was applied to further filter the final SNP dataset. The threshold used to detect heteroplasmies was 30% of mutated bases and heteroplasmies with coverage lower than 20 were filtered out. Lastly, fasta were produced using HaploGrep2^47^, which was also employed to predict haplogroup for every subject. The sequence data are available in GenBank with the accession numbers (PV621465 - PV621837) and can be accessed via the following link: https://www.ncbi.nlm.nih.gov (Here is the link: **373 mitogenomes - Colombo**). Any additional information concerning sequence and analytical data employed in this paper is available from the lead contact upon request.

### Phylogenetic and phylogeographic analyses

The 373 North African and sub-Saharan African mitogenomes were analysed together with 495 modern and 43 ancient complete mitogenomes publicly available (Supplementary Tables S1 and S2). The sequences were compared to the rCRS^42^ using Sequencher 5.0 (Gene Codes Corporation). We built the phylogenetic tree by using a maximum parsimony approach. Haplogroup labels were assigned by following the nomenclature proposed by PhyloTree database build 17 (at http://www.phylotree.org/)^48^. Known problematic regions and mutations close to indels (from nucleotide positions (nps) 302 to 318 and from nps 3105 to 3110) were not considered in the phylogenetic tree^49^. Variant calls were further evaluated phylogenetically by assessing for each mitogenome the congruence with the expected haplogroup background^48^. By using this approach, potentially incorrect calls were identified, and eventually corrected.

The frequencies of macro-haplogroups and haplogroups were evaluated across the entire dataset of modern and ancient mitogenomes analysed in this study. Due to the small size of groups based on ethnic/linguistic affiliation, mitogenomes were grouped solely on geographic origin. A smaller phylogenetic tree, encompassing only North African-specific haplogroups (H1v, H1x, H1w and J2a2b1a*) was also built. These three H1 sub-lineages were previously described as North African-specific^50^while J2a2b1a* was considered as North African-specific as it fulfilled the requirements previously established by Olivieri et al.^49^: (i) it includes only mtDNAs of North African origin; (ii) it is defined by at least three mitogenomes, encompassing a minimum of two haplotypes (i.e. differing by at least one homoplasmic mutation); (iii) it is characterised by at least one stable mutation (i.e. not recurrent in the tree) at its root. A total of 40 subjects was employed for the construction of the tree, also including mitogenomes published in papers centred on the characterisation of specific lineages. Finally, in order to answer questions concerning a single clade, the phylogenetic tree of the L1b1a6 haplogroup was also built by including all publicly available mitogenomes. These datasets were also analysed using the software BEAST v. 2.5^51^ with the same settings reported below.

The entire dataset was analysed with the software Bayesian Evolutionary Analysis Sampling Trees (BEAST) 2.5^51,52^. The program ran was under the HKY substitution model (gamma-distributed rates plus invariant sites) with a fixed molecular clock for a minimum of 150,000,000 iterations, with samples drawn every 10,000 Markov chain Monte Carlo (MCMC) steps, after a discarded burn-in of 10,000,000 steps, as in Olivieri et al.^49^. All haplogroups and their major subclades were considered monophyletic in the analyses. Following the same approach as in Olivieri et al.^49^ the two clock rates proposed by Soares et al.^53^ and Posth et al.^54^ (2.3 ± 0.2 × 10^− 8^ and 2.7 ± 0.2 × 10^− 8^ base substitutions per nucleotide per year over the entire mitogenome, respectively) were entered as priors in separate runs. Radiocarbon dates of ancient specimens or the age estimate of the burial cultural context were used as priors in the analysis. The demographic analysis was performed by using Tracer v1.7.1^55^, the results were then plotted with the package *ggplot2*^56^ employing a generation time of 25 years.

### Principal component analysis

In order to graphically display (and summarise) the relationships among different populations, Principal Component Analysis (PCA) was performed. The North African mitogenomes analysed in this study, were compared to a dataset of around 18,000 mitogenomes, selected from western Eurasian and sub-Saharan African populations. Only data from population-based studies and available on public repositories (Supplementary Table S3) was used for this step of the analysis. The PCA was performed on haplogroup frequency and computed with *prcomp*^57^ command from the *stats* R package and plotted using *ggplot2* ^56^ and *factoextra*^58^.

## Results and discussion

**Fig. 1:**
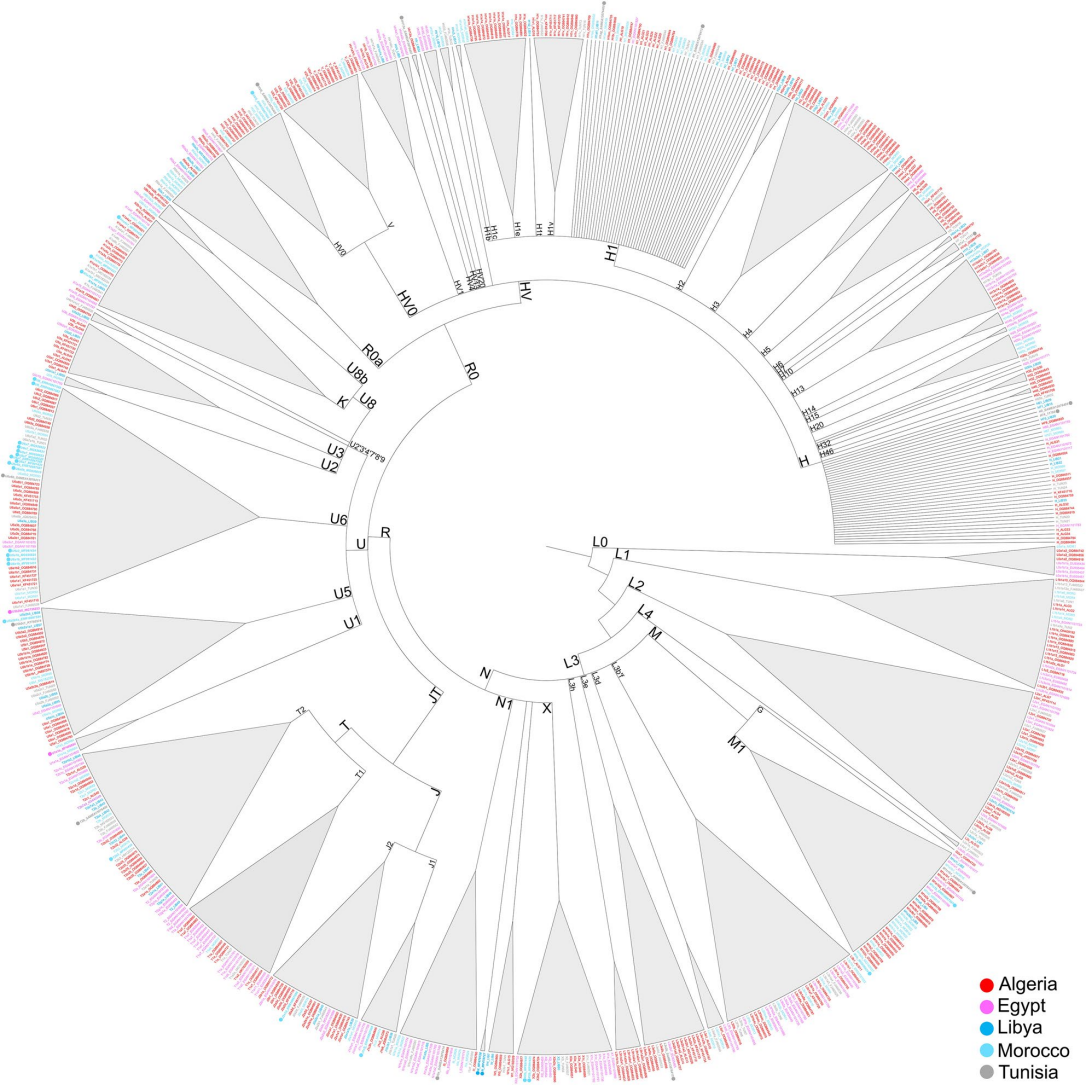
Schematic phylogenetic tree of modern (*N* = 733) and ancient (*N* = 43) mitogenomes from North Africa. Grey triangles correspond to different haplogroups and sub-haplogroups and their width is proportional to the number of included samples. Ancient subjects are indicated in bold italic and are flanked with a circular marker (see Supplementary Figure S1 for details).

We evaluated the mitogenome variation of 776 (733 modern and 43 ancient) North African subjects from Morocco, Algeria, Tunisia, Libya, and Egypt. The dataset, which includes 238 newly sequenced modern mitogenomes of North African origin together with 495 and 43 previously published mitogenomes from modern and ancient subjects, respectively (Table 1, Supplementary Tables S1 and S2), was employed to reconstruct a comprehensive phylogenetic tree of all available North African mitogenomes (Fig. 1, see Supplementary Figure S1 for details).

### Mitogenome variation in modern North Africa

**Fig. 2:**
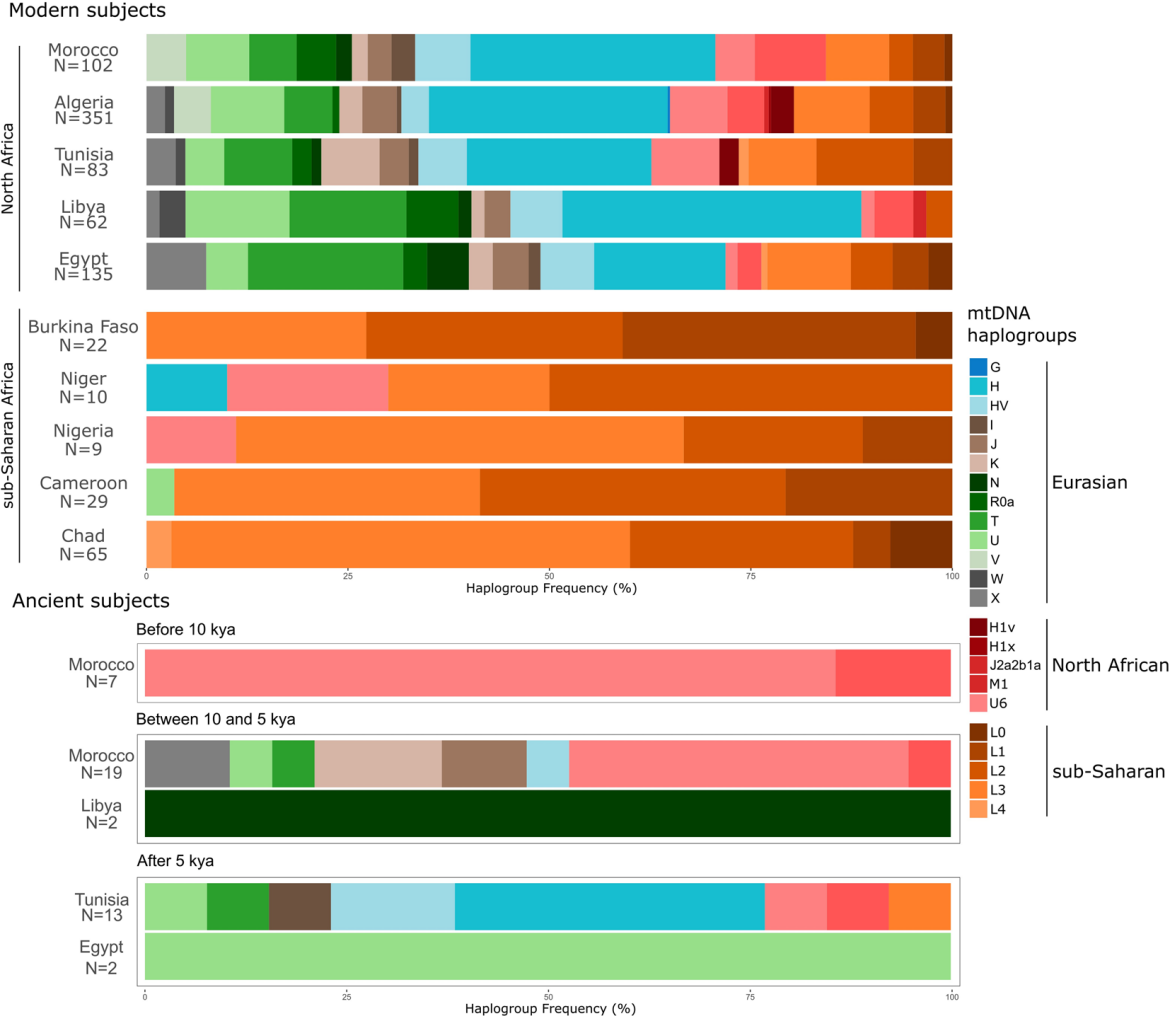
Barplots illustrating macro-haplogroup/haplogroup frequencies (as reported in Supplementary Table S4) in North African (Morocco, Algeria, Tunisia, Libya, and Egypt from west to east) and sub-Saharan (Burkina Faso, Niger, Nigeria, Cameroon, and Chad from west to east) modern and ancient mitogenomes analysed in this study. Ancient subjects are clustered into three groups based on their age estimates (before 10 kya, between 10 and 5 kya and after 5 kya). Haplogroups on the right are grouped according to their geographic origin/distribution.

The analysis revealed that modern North African mitogenomes (*N* = 733) cluster into 622 different haplotypes, showing a value of haplotype diversity of 0.9993, similarly to the results previously obtained on control-region sequences of Berber populations^30^. The high haplotype variability is also correlated with the identification of a high number of haplogroups and sub-haplogroups most of which (236 out of 368 identified clades) are members of typical western Eurasian branches (H, HV, I, J, K, N1, R0a, T, U, W, and X), observed at a frequency range between 62.6% (Tunisia) and 88.7% (Libya) (Fig. 2). A significant number of typical/autochthonous North African clades (45 out of 368, specifically H1v, H1x, M1, J2a2b1a* and U6 and their subclades) were also detected with a frequency range between 15.4% (Algeria) and 4.4% (Egypt). Lastly, sub-Saharan haplogroups (within macro-haplogroup L) encompass the remaining clades (87 out of 368) with frequencies spanning from 26.5% (Tunisia) to 3.2% (Libya) (Fig. 2, Supplementary Figure S1, Supplementary Tables S1 and S4). The frequency data of modern mitogenomes analysed and published ancient North African mitogenomes were compared in Fig. 2. However, the latter dataset, for the sake of clarity, will be discussed later.

**Fig. 3:**
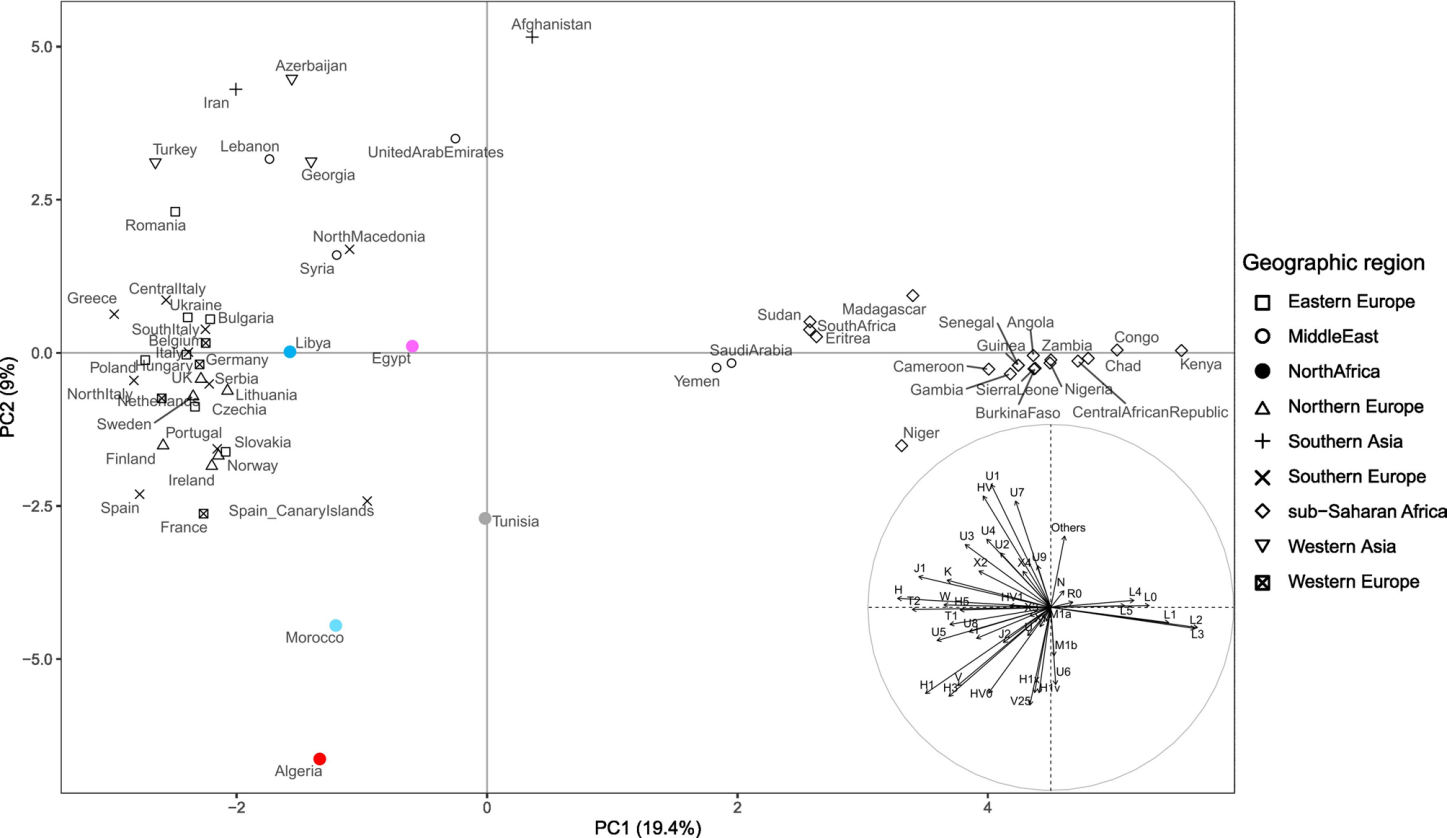
PCA plot showing the genetic variation of North Africa in the context of neighbouring sub-Saharan African and Western Eurasian landscapes. Data from sub-Saharan Africans are from the present study, while the Eurasian dataset includes over 18 thousand sequences available in public databases (Supplementary Table S3). The plot is based on modern mtDNA haplogroup frequencies from both this study and the literature. Reference populations are indicated by the country name, followed by a symbol to represent their macro-area. North African populations analysed in this study are highlighted in different colours: red (Algeria); pink (Egypt); blue (Libya); light blue (Morocco) and grey (Tunisia). The inset shows haplogroup contributions to PC1 and PC2 (accounting for 19.4% and 9.0% of the total variance, respectively).

Overall, the typical/autochthonous component accounts for a minor portion of North African modern mitogenomes, while the Eurasian one plays a major role, albeit with qualitative and quantitative differences from population to population. For this reason, the mtDNA gene pool of single North African populations may be closer to that of western Eurasian populations. This is well illustrated in the graph of the first two principal components shown in Fig. 3 where North African groups are located at various distances between western Eurasian populations without forming a distinctive cluster. The Eurasian component is the largest contributor to the North African variability, comprising more than half of the lineages described in the region (quadrants I and II of the inset plot, see Table S3 for details).

### The Eurasian component in North Africa

North African populations show high frequencies of haplogroups of Eurasian origin, that potentially diffused in this area at different times from three main sources: western Europe through the Gibraltar strait, the Middle East via the Sinai Peninsula, and the Mediterranean basin by sea. The five North African populations analysed in our dataset confirmed this trend, with at least 68% of the mtDNA variability represented by Eurasian clades (Fig. 2; Supplementary Table S4). Considering that multiple sources contributed to the diffusion of these lineages in North Africa, the Eurasian component in its totality appears almost equally frequent from west to east (ranging from 62.6 to 71.8%), with the only exception being represented by the Libyan population, with the highest value of 88.7% of Eurasian clades (Fig. 2). However, if we take into consideration one by one the haplogroups that make up the Eurasian component, the scenario changes and we can observe four different trends of distribution: (i) haplogroups with frequencies decreasing from west to east; (ii) haplogroups with frequencies decreasing from east to west; (iii) haplogroups with frequency peaks in central North Africa and decreasing clines at both sides; and (iv) haplogroups equally represented in the whole area.

The first group is the most frequent in our dataset and includes haplogroups mainly diffused in western Europe, such as H (represented by more than 20 sub-clades in our dataset). In particular, H1 and H3 are observed with frequency peaks in western North Africa (Morocco or Algeria) and lower frequencies in the eastern edge of Egypt, except for Libya that, despite being in the centre of North Africa, shows a high percentage of both clades (9.7% and 4.8%, respectively). A similar trend was observed for haplogroups HV0 and V (including V25) that are present in Northwest Africa and not observed in Tunisia (V and V25), Libya, or Egypt (HV0). Haplogroups H (including H1 and H3), HV0 and V reached North Africa mainly via the Strait of Gibraltar, as suggested by their geographical distribution across Eurasia and as already envisioned by previous studies^59–61^.

The second group, with a frequency cline from east towards west, includes haplogroups HV1, with a peak of 2.9% in Egypt and not detected in Morocco, T, with 19.3% in Egypt and then decreasing towards west, and N1 (including both N1a and N1b), which reaches 5.2% in Egypt. Both HV1 and N1 most likely originated in the Near East^62–64^as well as haplogroup T, together with its sister clade J^65,66^and they all moved towards Europe, North Africa, and even sub-Saharan Africa (as for haplogroup N1^39^) during both pre- and Neolithic periods as well as in more recent times. Haplogroup T includes a sub-clade, namely T1a7, which was previously identified as typical of North African regions^40^. In our comprehensive phylogeny (Supplementary Figure S1), it encompasses eight mitogenomes (six from Egypt^34,67^ and two from Algerian Berbers^40^) and represents a direct link between the Near East and North Africa, due to the detection of mitogenomes in the Arabian Peninsula, Israel, Iraq and Lebanon descending from the clade root. Haplogroup X, despite being quite rare in Europe, reaches significant frequencies in North Africa, especially in Egypt, where it is present in the 7.4% of the population, with a decline towards west, with 3.6% in Tunisia and 2.3% in Algeria, but it was not observed in Morocco and shows an intermediate frequency of 1.6% in Libya. Finally, the rare haplogroups I and W are thought to have a similar history to the much more frequent J and T^68^even if haplogroup I is particularly represented in Morocco (2.9%).

The third group includes haplogroup K, which shows its highest frequency in Tunisia (7.2%) and a decline at both eastern and western sides. A similar trend can be observed also for haplogroup U (including all its sub-clades from U1 to U8 with the exception of the North African U6), with a peak of 9.1% in Algeria and a slight decline in the other regions, again with the exception of Libya, showing the highest value of 12.9%, mainly due to an excess of the characteristic European haplogroup U5^69^.

The fourth group of haplogroups, identified by those equally represented in the whole North African region, is not well represented in our dataset, with only haplogroup J ranging from about 3–4.4% in western, central and eastern North Africa. Haplogroup R0a, originated in the Arabian Peninsula, is most frequent in Arabia and the Horn of Africa^70^and is also present in our dataset al.though it does not show a frequency cline from east to west, as previously reported^70^. Indeed, although it reaches a frequency peak of 6.4% in Libya, it maintains significant occurrences in all the regions (4.9% in Morocco, 3% in Egypt, 2.4% in Tunisia and 0.9% in Algeria).

### The North African component

The North African mtDNA component includes two different types of clades. The first group is represented by haplogroups M1 and U6, which did not originate in North Africa, but are markers of a pre-glacial migration from southwest Asia^23–25,71^. They are both considered to be typical of North Africa, although M1 reaches peaks of frequency in the Horn of Africa and they are also distributed, at lower frequencies, throughout the entire Mediterranean basin. As already mentioned, their Palaeolithic presence in this area is also supported by the classification of ancient mitogenomes from Morocco (dated to 14.2–14.6 kya;^15,18,21^) into these haplogroups (six out of seven are U6 and one is M1, Supplementary Table S2). A basal haplotype of clade U6 was also found in two ancient individuals from Romania, dated back to around 34 − 33 kya, representing the legacy of the spread of the clade from western Asia into Eastern Europe^72,73^probably the European counterpart of the parallel spread to North Africa. Among the modern North African mitogenomes analysed in this study, haplogroup U6 was observed at frequencies ranging from 1.6 to 8.4% (4.9% in Morocco, 7.1% in Algeria, 8.4% in Tunisia, 1.6% in Libya and 1.5% in Egypt), in full agreement with previous data^24^. As for haplogroup M1, it was observed as M1a in Morocco (2.9%), Algeria (2.3%), Libya (4.8%) and Egypt (3%), and as M1b in Morocco (5.9%) and Algeria (2.3%).

**Fig. 4:**
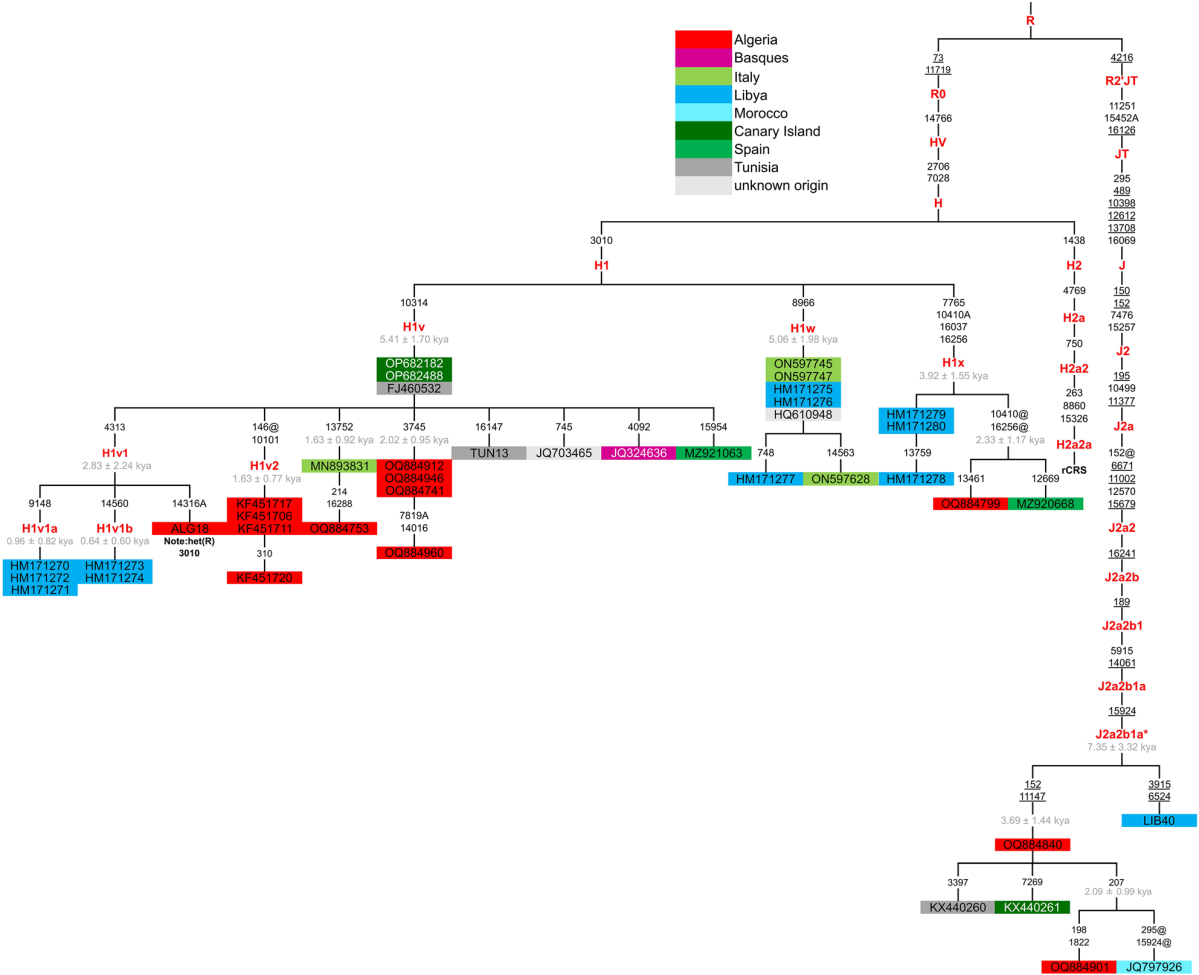
Phylogenetic tree of the North African-specific haplogroups H1v, H1x, H1w, and J2a2b1a* (included mitogenomes are listed in Supplementary Table S6).

The second type of North African component consists of haplogroups that can be defined as North African-specific because they arose in situ and their distribution remained restricted to North African populations. This group includes three sub-clades of haplogroup H1, namely H1v, H1x and H1w^50^and J2a2b1a*, a sub-clade within haplogroup J2^40^, all representing rare lineages. To shed light on the origin and dissection of these North African-specific clades, we built a phylogenetic tree encompassing all available mitogenomes (Fig. 4). The list of included mitogenomes is reported in Supplementary Table S6. Within H1 sub-clades, haplogroup H1v is the most frequent with 23 mitogenomes, including ten from Algeria, five from Libya and two from Tunisia. Together with these mitogenomes of confirmed North African origin, the clade also includes one mitogenome of unknown origin and five additional sequences that can be attributed to recent gene flow from North Africa, i.e. two mitogenomes from the Canary Islands, whose population originally moved from North Africa and is still characterised by a strong component from this source^74^, a Spanish subject, an Italian individual of Sephardic Jewish origin and one Basque individual. Haplogroup H1w includes seven mitogenomes, of which three from Libya, three from Italy and an additional sample of unknown origin, while haplogroup H1x encompasses five mitogenomes, three from Libya, one from Algeria and one from Spain. H1v and H1w show very similar coalescence ages (5.4 and 5.1, respectively), while H1x is slightly younger with a coalescence age of 3.9 kya, which largely overlap with the others when error is taken into account. This suggests that concomitant events of in situ differentiation occurred. As for the fourth North African-specific haplogroup, namely J2a2b1a*, it appears to be even rarer than the other three, comprising only six mitogenomes (two from Algeria and four from the Canary Islands, Morocco, Tunisia and Libya), but older, being dated back to about 7.4 kya.

The geographic distribution of the four North African-specific clades, which covers virtually all North African regions from west to east, does not allow to hypothesise specific places of origin in North Africa, even if haplogroup J2a2b1a* bears a mitogenome from Libya directly descending from its root with a unique haplotype. Additionally, because North African regions have experienced continuous gene flow from various Eurasian sources at different times, it is difficult to determine the timing and geographical distribution of nearby upstream nodes in the phylogeny and challenging to identify the origin and timing of the differentiation event that occurred in situ. With these considerations in mind, we evaluated the upstream node in the phylogeny, from which the closest external mitogenomes (out of North Africa) radiate. For the three H1 sub-haplogroups, the closest external mitogenomes are all those directly descending from the root of clade H1, thus counting a very high number of sequences and covering the entire areal of distribution of H1, the most represented H sub-clade across western and Mediterranean Europe. These data suggest that the founder haplotypes of the three H1 sub-clades arrived from Western/Mediterranean Europe and were already present in North Africa by at least 4–5 kya.

As for J2a2b1a*, the situation is different: only a single mitogenome belonging to an individual from southern Italy (Calabria region) is directly descended from the root of the upstream node defining sub-clade J2a2b1a, suggesting a direct link between the Mediterranean basin and this latter North African specific clade. However, for the reasons explained above, we cannot exclude that both the Italian mitogenome and the North African-specific clade J2a2b1a* share a common ancestor that was already present in North Africa or the Middle East before their differentiation (at least 7 kya), since both these regions are the home of the mitogenomes descending from the more upstream nodes of J2a2b1. Furthermore, as a general observation, the four North African-specific sub-haplogroups, except for H1v, encompass a relatively small number of mitogenomes, and, in the case of H1w, they are often characterised by the same haplotype, so that their estimated ages should be treated with some caution.

### The sub-Saharan African component in North Africa

The sub-Saharan contribution, defined by the sub-Saharan L lineages, was observed in all North African regions (Fig. 2, Supplementary Table S4), at different frequencies, without a specific trend in a north-south or east-west direction. Indeed, macro-haplogroup L reaches frequencies of 26.5% in Tunisia, 23.7% in Egypt, 19.7% in Algeria, and 15.7% in Morocco. Macro-haplogroup L includes five clades in North African populations, i.e. haplogroups L0, L1, L2, L3 (excluding haplogroups M and N) and L4 (Fig. 2), with L2 and L3 being the most frequent (as previously reported by^75^). Haplogroup L2 is widely distributed across North Africa (12% in Tunisia, around 5–6% in Algeria and Egypt and around 3% in Libya and Morocco), as already observed^30,76^. Haplogroups L3 and L1 (present in Algeria, Egypt, Morocco, and Tunisia with frequency ranges of around 8–10% and 4–5%, respectively) are also common in Libya (as already observed in^31^although absent in our sample). As expected, due to their sub-Saharan origin, all the five lineages within macro-haplogroup L mentioned above (Fig. 1; Supplementary Figure S1) encompass mitogenomes not only from North Africa, but also from the sub-Saharan African countries included in this study (i.e. Burkina Faso, Cameroon, Chad, Niger, and Nigeria). Within haplogroup L1, L1b1a6 (Supplementary Figure S2, Table S7), defined by two transitions at nps 9755 and 14,110, encompasses ten mitogenomes in our phylogeny, most of which are from west-central Africa (two from Burkina Faso, one from Nigeria and four from Cameroon), two from Morocco and one from Tunisia. This clade has been considered an example of direct input from the African continent to Europe/Iberia via North Africa^77,78^. We re-evaluated this hypothesis in light of our new data, building a phylogenetic tree with all available L1b1a6 mitogenomes (Supplementary Figure S2 and Table S7). We confirmed that haplogroup L1b1a6, dated back to almost 14 Kya, is distributed in western Africa, including mitogenomes from western sub-Saharan countries (i.e. Burkina Faso, Cameroon, Guinea-Bissau, and Nigeria) and from Morocco and Tunisia in North Africa. The European mitogenomes contained in clade L1b1a6 do not form a separate subclade, but they all descend from a node dated back to around 13 Kya, thus setting this date as the upper limit to the initial migration event, similarly to what previously proposed by Hernandez et al.^78^.

### Mitogenome variation in adjacent sub-Saharan regions

The high genetic variability of North Africans, measured in terms of number of haplogroups observed, is particularly evident when comparing North African mitogenomes with sub-Saharan sequences (from Burkina Faso, Niger, Nigeria, Cameroon and Chad) (Fig. 2, Supplementary Table S2). Indeed, most of the subjects from these countries belong to macro-haplogroup L, with few exceptions represented by one Nigerian mitogenome belonging to haplogroup U6b, two mitogenomes from Niger belonging to two distinct sub-haplogroups within U6a (U6a3b1 and U6a5a) and one U5b1b1b sample identified in a Fulbe from Cameroon. As mentioned above, haplogroup U6 is a typical North African clade, with frequency peaks in the north-western part of North Africa, while the U5b1b1b clade was already identified in Berber populations, as a marker of diffusion events from the Iberian Peninsula^69^.

### Mitogenome variation in ancient North Africa

Haplogroup distribution was also investigated for comparison in a dataset of 43 ancient published mitogenomes from North African populations (Fig. 2, Supplementary Tables S2 and S5). The ancient samples are from four countries (Morocco, Tunisia, Libya, and Egypt), spanning a time frame of about 13 ky.

The most ancient mitogenomes, represented by seven samples, are from the Iberomaurusian site of Taforalt in Morocco, which date back to 15 kya and constitute the oldest North African DNAs^18^. One mitogenome belongs to haplogroup M1b, while the remaining six classify into different U6a sub-clades, thus confirming the Palaeolithic origin of the two typical North African haplogroups M1 and U6 (Fig. 2). Nineteen additional unrelated ancient mitogenomes are available from Moroccan archaeological sites, from Epipalaeolithic and Neolithic cultures, with dates ranging from ~ 7 kya to ~ 4 kya^15,21^. Eight U6 (six of them U6a) and one M1a mitogenomes were detected in Epipalaeolithic, Early Neolithic and two (out of three) Middle Neolithic samples, while the remaining mitogenomes including all the Late Neolithic ones belong to both European and Near Eastern clades, specifically HV0, J1c, J2a, K1a, T2b3, U5b, and X2b. These overall data confirm the presence of an ancient North African genomic component with an ultimate source in Upper Palaeolithic Eurasia, as previously suggested by modern dataset^23,24^ (albeit possibly via several waves^71^). This input was followed by Neolithic gene flow from western Europe and subsequent flow from the Middle East starting in the Middle Neolithic^15,21^. These details are supported more generally by genome-wide analyses^15,21^.

From a Neolithic site in Libya’s central Sahara (dated to ~ 7 kya), two mitogenomes descending from haplogroup N were identified^64^. All the remaining 15 ancient individuals available from North African regions date back to more recent times (from 4 kya to 1.3 kya), and were collected from Tunisia (from two Phoenician sites)^20,79^ and Egypt^80–82^. Notably, among these complete ancient genomes, only three from the Phoenician site of Kerkouane belong to haplogroups M1, U6 and L3e^20^ while all the others classify within haplogroups of Eurasian origin (H, HV, I5, T2, and U) (Fig. 2).

However, it is important to note that of the 43 available ancient samples in North Africa, 39 are from the western regions (26 from Morocco and 13 from Tunisia), with just two samples available from Libya and two from Egypt. As mentioned in the introduction, climatic conditions in Africa are generally not favourable for the preservation of ancient DNA, and these studies are generally affected by mosaic sampling. Expanding the sampling to eastern regions of North Africa would therefore be mandatory in order to obtain a broad picture of the ancient genomics of the region.

### The mitogenome links between North Africa and Sardinia

In 2017, a study of mitogenomes from Sardinia identified a large number of autochthonous sub-haplogroups that arose in situ, in some cases shortly after the first peopling event of the island and were defined as Sardinian-specific since their geographic distribution was apparently restricted to the Mediterranean island^49^. By comparing the phylogeny of North African regions with that based on Sardinian-specific haplogroups, we identified a connection between North Africa and Sardinia involving at least four sub-clades. Indeed, the haplogroups H1cn1a, H3aw, H3ay1, and J1c3h, previously defined as Sardinian-specific, now include not only Sardinian mitogenomes but also one or few mitogenomes from our North African dataset.

These North African mitogenomes most likely reveal temporally distinct events of female-mediated gene flow from Sardinia to different regions of North Africa. In detail, the Sardinian-specific haplogroup H1cn1a includes also one mitogenome from Morocco that differs from other Sardinian mitogenomes by only one mutation (np 16162) in the control region. This most likely represents a very recent connection between the two regions, dated back to only a few hundred years, given the low molecular divergence accumulated within the clade. The H3aw haplogroup, a subclade of H3 defined by the transition at np 15,289, includes three identical mitogenomes from Algerian Berbers harbouring the control-region transition at np 194 from the H3aw root. This connection could possibly be further back in time, as the haplotype found in the three Berbers departs from the root of H3aw that had been dated to around 3000 years ago^49^. The Sardinian-specific haplogroup H3ay1 shows a mitogenome from Libya with a haplotype corresponding to the clade root itself and identical to a sample from southern Sardinia. This too might be a rather ancient link, as the clade root was dated to around 3400 years ago. However, in both cases, recent events of gene flow cannot be excluded. Finally, an Algerian Berber has a mitogenome descending from the J1c3h haplogroup root, dated 5400 years ago^49^with three mutations, the reversion of a transition in the control region and two transitions in the coding region.

Another interesting case concerns the H3u haplogroup, which was not classified as Sardinian-specific, but contains only Sardinian and North African samples, including three mitogenomes in our phylogeny (from Algeria, Libya and Morocco). It is worth noting that an ancient Sardinian sample^49^ dated back to the Final Bronze Age (4000 years ago) and from the south of the island, belongs to the same clade and shares the mutation at np 16,240 present in all three North African mitogenomes of this study, again indicating a link between the two regions that most likely goes back several millennia. Finally, as previously noted^40^two mitogenomes from Algeria share the same W5 haplotype with a Punic-era Sardinian individual from the late 5th century^83^.

On the other hand, if we consider the possible connections between North African regions, Sardinia and one or more Mediterranean regions, the examples are too numerous to list. To give just one example among many, haplogroup H1e1a6 includes two mitogenomes of Moroccan origin, produced in this work, together with an ancient Sardinian sample from the Punic era^83^ and modern Spanish mitogenomes^84,85^.

### The demography of North African populations over time and a signal from the last green Sahara

**Fig. 5:**
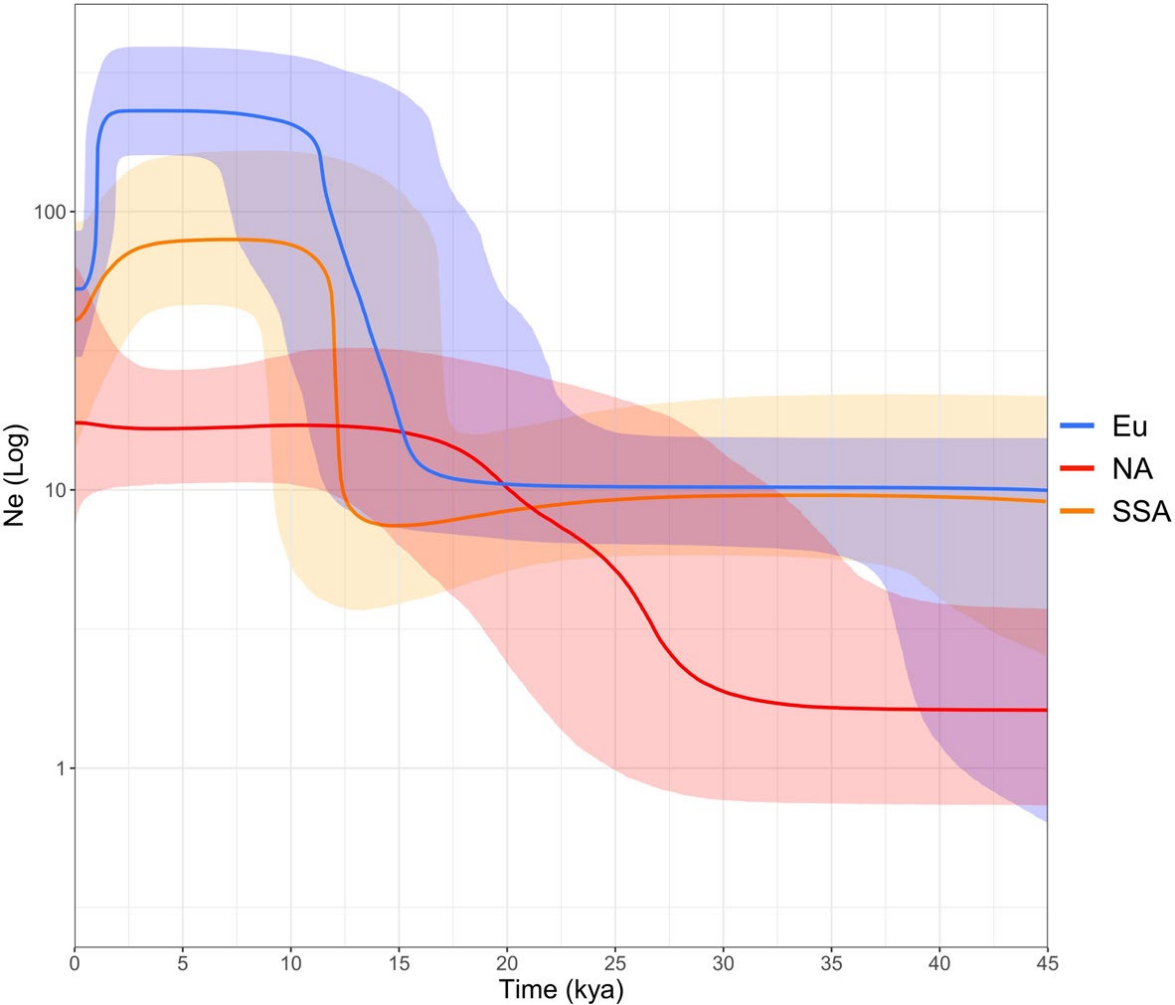
Bayesian Skyline Plot showing the effective population size trends (Ne) of haplogroups found in the dataset of 733 modern and 43 ancient mitogenomes from North Africa. Haplogroups were divided into three groups, according to the three genetic components (as reported in Supplementary Tables S4 and S5) contributing to the North African gene pool, Eu (Eurasian), NA (North African) and SSA (sub-Saharan African). Solid lines are the median estimates obtained by employing the mutation rates proposed by Soares et al.^53^; the shadings show the highest posterior density limits.

In order to outline the complexity of mtDNA sequence variability in North African populations, observed in both ancient and modern times, we performed a Bayesian analysis. The Bayesian Skyline Plot (BSP) in Fig. 5 shows changes in the effective population size over time for mtDNA lineages identified in North African populations. The same analysis was performed by using two different clock rates^53,54^ and the results are virtually overlapping. As discussed previously, North African maternal genetic components can be divided into three groups of distinct geographic origin, i.e. sub-Saharan, North African typical/autochthonous and Eurasian (as illustrated in Supplementary Tables S4 and S5). These three components trace their origins to different places and different times. For this reason, we performed coalescent Bayesian skyline analyses employing the entire dataset of modern (*N* = 733) and ancient (*N* = 43) mitogenomes from North African populations, partitioned according to these three components (Fig. 5).

Each component reveals a distinct major population growth event. The expansion of the Eurasian component (blue line) begins in pre-Neolithic times, at ~ 17.5 kya, corresponding to the retreat of the glaciation in Europe. This result indicates that along with the Neolithic impact that brought Eurasian lineages into North Africa, as also attested by the available genomic and ancient DNA data^4,15,16,86^a pre-Neolithic expansion also left traces in the Eurasian mtDNA variability in North African populations, although this expansion most likely took place in Europe or western Asia before the arrival in North Africa. Considering the age and geographical distribution of the haplogroups, it is likely that, for example, U5b1b arrived from the Strait of Gibraltar, while others may have arrived from the Sinai or the Mediterranean. The autochthonous/typical North African component (red line), by contrast, indicates an expansion beginning around 35 kya, from populations in the Middle East, as also previously hypothesised by mitochondrial and nuclear ancient and modern DNA data^18,24,87,88^. The discovery of putatively haplogroup pre-N lineages in early Holocene southwest Libya^64,89^which lack the N-defining 8701 A variant similar to Oase-1 from Palaeolithic Romania, but carry the N-defining 9540T that Oase-1 also lacks^73^, may point to the same process. On the other hand, the 60% Natufian-related ancestry in the Libyan samples may suggest a more recent wave of Middle Eastern ancestry.

The sub-Saharan component (orange line), by contrast with both, shows a primary event of expansion between 15 and 10 kya. This time span overlaps with the last Green Saharan period, which occurred 15 to 5 kya and was characterised by human settlements throughout the Sahara. Y-chromosome and genomic data supported the scenario of trans-Saharan movements towards North Africa over a time span of ~ 12 − 5 kya mediated by a ghost population they called the Green Sahara Population^90,91^. As for the female-mediated mtDNA, a signal of gene flow across the Sahel belt during the Holocene was proposed from founder analyses^92^ on the HVS-I variability in African populations. In particular, these previous data indicated a signal of expansion from sub-Saharan Africa towards North Africa with a peak at around 6.5 Kya, attributable mostly to haplogroups L1b (L1b1a in particular), L3e5, and L0a1^92^, albeit potentially biased by primarily haplogroup-based sampling at the time.

Here, BSP analysis with a much larger dataset, based on both modern and ancient mitogenomes from North Africa provides further evidence that the Green Sahara may have played an important role in shaping the genetic landscape of North African populations also from a female-mediated perspective.

This signal is present in sub-haplogroups of all sub-Saharan lineages (L0 to L5), suggesting that the sources from which these movements originated may be multiple. It is worth mentioning that the limited amount of genomic data available from both modern and ancient populations from sub-Saharan Africa makes it difficult to identify and date the specific lineages that might be responsible for the observed signal. However, we can hypothesize a few possible examples. Haplogroup L2a1 is observed in modern subjects from both sides of the Sahara Desert and in ancient Sudanese^19^but also in ancient samples from Andalusia dated 3.5 kya^93^and in two samples from Sardinia (2.5–1.7 kya)^94^thus suggesting that this lineage arrived in Europe prior to 3.5 kya and may have crossed the Mediterranean basin at a later date. The distribution of the L2a1 clade in modern North Africans is widespread throughout the region, suggesting that multiple events of gene flow occurred across the Sahara during the period of favourable climatic conditions in the Green Sahara. Similarly, as previously discussed (Supplementary Figure S2, Supplementary Table S7) haplogroup L1b1a6 includes sub-Saharan and North African, as well as European, mitogenomes descended from the same node with an upper limit set by the age of the L1b1a6 node at almost 14 thousand years ago.

Future analyses may increase the number of proxy populations from sub-Saharan Africa, providing a more accurate estimate of the times and routes of contact that shaped the formation of modern North African population groups.

## Conclusion

Since the 1990s, uniparental markers, i.e. the maternally-inherited mtDNA and the male-specific non recombining portion of the Y chromosome (MSY) have provided informative data for population geneticists to trace ancient human migrations at both macro- and micro-geographic levels^32,35,95,96^. In particular, mitochondrial DNA, with its small molecular size and high evolutionary rate, has long been a key genetic tool in population studies. It is important to emphasise that, compared to nuclear genes, mtDNA does not reflect the full complexity of past demographic processes. It is a single, maternally-inherited locus that is particularly susceptible to genetic drift due to its reduced effective population size. Nevertheless, this non-recombining genetic system can provide high phylogenetic and phylogeographic resolution^32^, making the mtDNA data an informative complement to nuclear genome data in the genomic era. In particular, large-scale surveys of complete mitogenomes allowed unprecedented resolution of the mtDNA phylogeny (in terms of haplogroup and sub-haplogroup definitions). Phylogenetic and phylogeographic approaches have allowed accurate dating and calibration of the mtDNA molecular clock^53,97^, useful also as a stand-point for dating demographic events detected through genomic data^98^.

After an initial phase focusing on the mitogenomes (or portion of mtDNA) variability of specific phylogenetic lineages and assessing it on their entire distribution range, population mitogenomics moved towards the analysis of populations living in restricted geographical areas, through fine-scale and massive surveys of mitogenomes, in order to identify all the specific genetic components and lineages characteristic of that population^49^. One geographical region whose mitogenomic variability had never been studied using a population-based approach is North Africa. North Africa occupies a unique geographical position in the southern coast of the Mediterranean basin, has another geographical barrier to the south in the form of the Sahara Desert, and has two main routes of communication with the Middle East to the east via the Sinai Peninsula and with Europe to the west via the Straits of Gibraltar. For this reason, studying the genetic variability of this geographical region means looking for signs of admixture with multiple peoples who have contributed to its gene pool over the millennia.

**Fig. 6:**
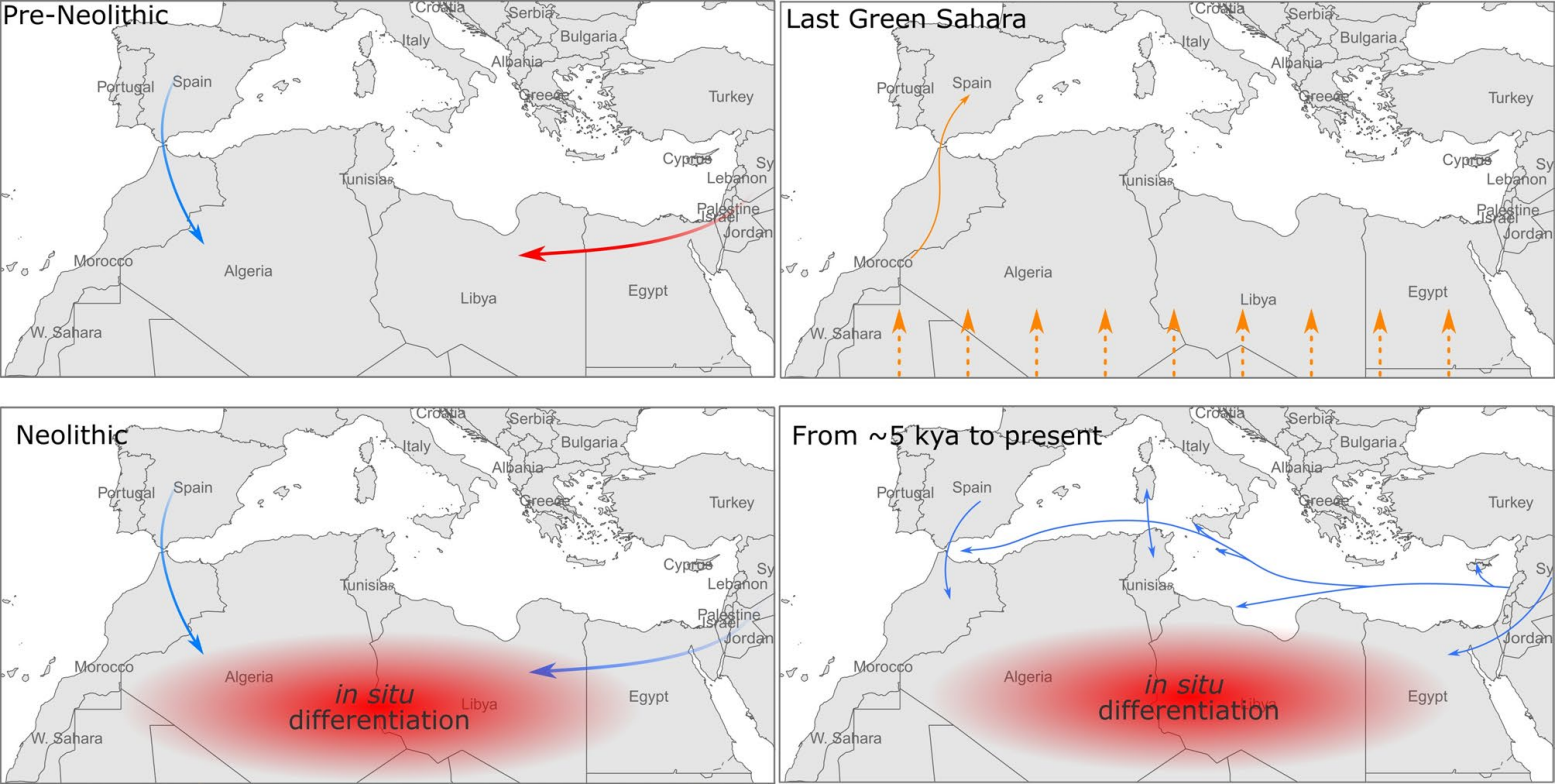
Representation of the major movements of the three maternal genetic components reported in this study. The colours of the arrows are in agreement with the three genetic components identified (Eurasiatic -blue-, sub-Saharan -orange- and North African -red-). The underlying map was built using the R package *rnaturalearth v.1.1.0 (*https://docs.ropensci.org/rnaturalearth/^101^*)*.

In this paper we have analysed and discussed a dataset of 733 modern (238 of which new) and 43 ancient mitogenomes from Morocco, Algeria, Tunisia, Libya and Egypt, in comparison with 17,274 modern mitogenomes, 11,438 from Eurasia and 5,836 from sub-Saharan African countries (135 of which new). Our analyses, based on a phylogeographic approach, comparisons with population (and non-population) data on both modern and ancient mitogenomes, and demographic investigation using a Bayesian approach, in the light also of the available genomic data, suggest that the mitogenome variability in North African populations (i) can be traced back to three main origins - Eurasia, North Africa and Sub-Saharan Africa - and (ii) has been defined during four key time periods. The three components mainly include haplogroups that arrived in North Africa through movements and migrations, from different sources and at different times, as well as a small proportion of haplogroups that differentiated in situ, also at different times. As shown in Fig. 6, the first key events defining the North African genetic pool occurred in the Palaeolithic, with the arrival from western Eurasia of a characteristic North African component characterised by haplogroups M1 and U6.

Two major routes, through the Strait of Gibraltar and Sinai, were followed during the emergence of Neolithic culture, which brought numerous subclades from both west and east as supported by genomic data from both modern and ancient samples^4,15,16,21,86,91^. The typically sub-Saharan African component (L sub-clades) found in North African populations seems to have come from the south mainly during the favourable climatic period of the Green Sahara. This corridor of human movements, involving a very large area and probably several population sources, has also been identified by modern genomic and Y-chromosome data^90,92^; the data presented here emphasises that it appears to have been not only a male-mediated event, but also left traces in the maternally inherited mtDNA. Once they reached North Africa, some sub-lineages of haplogroup L, such as L1b1a6, entered Europe via the Iberian Peninsula (as suggested by^78^).

In more recent times, North Africa has been involved in a myriad of exchanges with peoples from neighbouring regions and the wider Mediterranean basin. Figure 6 shows just a few of the many possible exchange routes and indicates a period from about 7.5 kya, corresponding to the coalescence ages of J2a2b1a*, which differentiated in situ in North Africa^40^. Other in situ differentiation events occurred in more recent times, starting from about 5 kya (Fig. 4), giving rise to the North African autochthonous haplogroups H1v, H1w and H1x ^50^. From the Bronze Age until a few centuries ago, trade and exchange between peoples strongly contributed to North African mtDNA variability. Our data show matrilineal links between Sardinia and North Africa, and between these two regions and others, such as Spain, in the Mediterranean basin. Trade between the Iberian Peninsula and North Africa has also been constant over time^99^. Furthermore, previous studies^63,83^ have reported how trade and/or invasions by peoples such as the Phoenicians and the Vandals left extensive genetic traces, supporting the hypothesis of extensive female movements throughout the Mediterranean, although it is not yet clear what social dynamics were involved in this process. Other routes, such as the Arab invasion, particularly from the 7th century AD onwards, have been suggested from the spread of Y-chromosome haplogroups^100^ and are likely to have significantly reshuffled and modified the genetic variability of North African populations, although a larger dataset, possibly including more Berber populations, should be analysed to provide evidence from a matrilineal perspective. For a clearer picture, it will be necessary to further increase the number of both modern and ancient genomes from North Africa, which is still poorly represented in terms of sequenced genomes in comparison with other parts of the world.

## Electronic supplementary material

Below is the link to the electronic supplementary material.

Supplementary Material 1

Supplementary Material 2

## Data Availability

The sequence data of the paper “The origin of modern North Africans as depicted by a massive survey of mitogenomes” are available in GenBank with the accession numbers (PV621465 - PV621837) and can be accessed via the following link: https://www.ncbi.nlm.nih.gov.
