# Supplementary material for: The origin of modern North Africans as depicted by a massive survey of mitogenomes
Source: Sci Rep. 2025 Jul 25;15:27025. doi: 10.1038/s41598-025-12209-x (PMC12289972; doi:10.1038/s41598-025-12209-x)
Supplement: Supplementary file 1 — Supplementary Material 1 [file 41598_2025_12209_MOESM1_ESM.docx]

**Supplementary information**

**The genetic picture of North Africa as depicted by a massive survey of mitogenomes**

Giulia Colombo^1^, Elisabetta Moroni^1^, Alessandro Raveane^1^, Nicola Rambaldi Migliore^1^, Vincenzo Agostini^1^, Rosalinda Di Gerlando^2^, Claudio Fiorini^3^, Leonardo Caporali^3^, Francesca Gandini^4,5^, Elena Raimondi^1^, Eugenia D’Atanasio^6^, Hovirag Lancioni^7^, Valerio Carelli^3,8^, Maria Pala^9^, Beniamino Trombetta^10^, Andrea Novelletto^11^, Jean-Michel Dugoujon^12^, Alessandro Achilli^1^, Antonio Torroni^1^, Martin B. Richards^9^, Fulvio Cruciani^10^, Ornella Semino^1^, Anna Olivieri^1,*^

^1^Department of Biology and Biotechnology “Lazzaro Spallanzani”, University of Pavia, 27100 Pavia, Italy.

^2^Molecular Biology and Transcriptomic Unit, IRCCS Mondino Foundation, Pavia, Italy.

^3^IRCCS Istituto delle Scienze Neurologiche, Programma di Neurogenetica, 40139 Bologna, Italy.

^4^B‐Cell Neoplasia Unit and Strategic Research Program on CLL, IRCCS Ospedale San Raffaele, Milan, Italy.

^5^Medical School, Università Vita‐Salute San Raffaele, Milan, Italy.

^6^Istituto di Biologia e Patologia Molecolari (IBPM), Consiglio Nazionale delle Ricerche (CNR), 00185 Rome, Italy.

^7^Department of Chemistry, Biology and Biotechnology, University of Perugia, 06123 Perugia, Italy.

^8^Department of Biomedical and Neuromotor Sciences, University of Bologna, 40139 Bologna, Italy.

^9^School of Applied Sciences, University of Huddersfield, Queensgate, Huddersfield, HD1 3DH.

^10^Dipartimento di Biologia e Biotecnologie "Charles Darwin", Sapienza Università di Roma, 00185 Rome, Italy.

^11^Department of Biology, University of Rome "Tor Vergata", 00133 Rome, Italy.

^12^Laboratoire d'Anthropologie Moléculaire et Imagerie de Synthèse (AMIS), UMR 5288, Université Paul Sabatier Toulouse III, 31073 Toulouse, France.

*Corresponding author. E-mail: anna.olivieri@unipv.it.

**Supplementary Figure S1.** Maximum-parsimony phylogenetic tree of 884 modern (511 previously published) and 105 ancient mitogenomes from North and sub-Saharan Africa. All the samples are coloured according to their geographic origin as shown in the legend, while the grey lettering indicates ancient subjects. Sub-haplogroups defined for the first time in this study are circled in red. Mutations are shown on the branches (relative to rCRS); they are transitions unless the base change is explicitly indicated. The suffix @ indicates the reversion of a mutation occurring earlier in the phylogeny. Heteroplasmies are indicated with the prefix “het”. Recurrent mutations in the tree are underlined. Newly described haplogroups are indicated with red circles. ID numbers of mitogenomes correspond to those in Supplementary Tables S1 and S2.

**Supplementary Figure S2.** Maximum-parsimony phylogenetic tree of 29 modern mitogenomes belonging to haplogroup L1b1a6. All the samples are coloured according to their geographic origin as shown in the legend. Mutations are shown on the branches (relative to rCRS); they are transitions unless the base change is explicitly indicated. The suffix @ indicates the reversion of a mutation occurring earlier in the phylogeny. Heteroplasmies are indicated with the prefix “het”. Recurrent mutations in the tree are underlined. Newly described haplogroups are indicated with red circles. ID numbers of mitogenomes correspond to those in Supplementary Tables S7.

**Supplementary Figure S3.** Bayesian skyline plot showing the effective population size trends (Ne) of haplogroups found in the dataset of 733 modern and 43 ancient mitogenomes from North Africa. The BSP was performed as in Figure 5, but employing the mutation rate proposed by Posth et al.^95^. Solid lines are the median estimates, while the shadings show the highest posterior density limits.

**Supplementary Table S1.** Origin and sub-haplogroup affiliation of the 884 modern mitogenomes (373 newly sequenced) from North and sub-Saharan Africa analysed in this study.

**Supplementary Table S2.** Origin and sub-haplogroup affiliation of the 105 ancient mitogenomes from North Africa analysed in this study.

**Supplementary Table S3.** Frequency (in %) of haplogroups observed in modern mitogenomes from North (N=749) and sub-Saharan Africa (N=135) analysed in this study. Colours in the first column indicate the haplogroups’ origin.

**Supplementary Table S4.** Frequency (in %) of haplogroups observed in ancient mitogenomes from North Africa (N=105) analysed in this study. Colours in the first column indicate the haplogroups’ origin.

**Supplementary Table S5.** Origin and sub-haplogroup affiliation of 40 modern mitogenomes belonging to North African specific haplogroups.

**Supplementary Table S6.** Haplogroups' frequencies of modern mitogenomes from Europe, Middle East, North and sub-Saharan Africa, used to construct a PCA plot (Figure 4).

**Supplementary references**

1. Aizpurua-Iraola, J., Abdeli, A., Benhassine, T., Calafell, F. & Comas, D. Whole mitogenomes reveal that NW Africa has acted both as a source and a destination for multiple human movements. *Sci Rep* **13**, 10395 (2023).

2. Costa, M. D. *et al.* Data from complete mtDNA sequencing of Tunisian centenarians: testing haplogroup association and the “golden mean” to longevity. *Mech Ageing Dev* **130**, 222–226 (2009).

3. Font-Porterias, N. *et al.* The genetic landscape of Mediterranean North African populations through complete mtDNA sequences. *Ann Hum Biol* **45**, 98–104 (2018).

4. Kujanová, M., Pereira, L., Fernandes, V., Pereira, J. B. & Černý, V. Near Eastern Neolithic genetic input in a small oasis of the Egyptian Western Desert. *Am J Phys Anthropol* **140**, 336–346 (2009).

5. Lippold, S. *et al.* Human paternal and maternal demographic histories: insights from high-resolution Y chromosome and mtDNA sequences. *Investig Genet* **5**, 13 (2014).

6. Pagani, L. *et al.* Tracing the route of modern humans out of Africa by using 225 human genome sequences from Ethiopians and Egyptians. *Am J Hum Genet* **96**, 986–991 (2015).

7. Serra-Vidal, G. *et al.* Heterogeneity in Palaeolithic population continuity and Neolithic expansion in North Africa. *Curr Biol* **29**, 3953-3959.e4 (2019).

8. Fregel, R. *et al.* Ancient genomes from North Africa evidence prehistoric migrations to the Maghreb from both the Levant and Europe. *Proc Natl Acad Sci USA* **115**, 6774–6779 (2018).

9. Loreille, O. *et al.* Biological sexing of a 4000-Year-old Egyptian mummy head to assess the potential of nuclear DNA recovery from the most damaged and limited forensic specimens. *Genes* **9**, 135 (2018).

10. Matisoo-Smith, E. A. *et al.* A European mitochondrial haplotype identified in ancient phoenician remains from Carthage, North Africa. *PLoS One* **11**, e0155046 (2016).

11. Molto, J. E. *et al.* Complete mitochondrial genome sequencing of a burial from a Romano–Christian cemetery in the Dakhleh Oasis, Egypt: preliminary indications. *Genes* **8**, 262 (2017).

12. Moots, H. M. *et al.* A genetic history of continuity and mobility in the Iron Age central Mediterranean. *Nat Ecol Evol* **7**, 1515–1524 (2023).

13. Simões, L. G. *et al.* Northwest African Neolithic initiated by migrants from Iberia and Levant. *Nature* **618**, 550–556 (2023).

14. Vai, S. *et al.* Ancestral mitochondrial N lineage from the Neolithic ‘green’ Sahara. *Sci Rep* **9**, 3530 (2019).

15. van de Loosdrecht, M. *et al.* Pleistocene North African genomes link Near Eastern and sub-Saharan African human populations. *Science* **360**, 548–552 (2018).

16. Mallick, S. *et al.* The Simons Genome Diversity Project: 300 genomes from 142 diverse populations. *Nature* **538**, 201–206 (2016).

17. Hartmann, A. *et al.* Validation of microarray-based resequencing of 93 worldwide mitochondrial genomes. *Hum Mutat* **30**, 115–122 (2009).

18. Maász, A. *et al.* Phenotypic variants of the deafness-associated mitochondrial DNA A7445G mutation. *Curr Med Chem* **15**, 1257–1262 (2008).

19. Malyarchuk, B. *et al.* Whole mitochondrial genome diversity in two Hungarian populations. *Mol Genet Genomics* **293**, 1255–1263 (2018).

20. Bianco, E. *et al.* Recent common origin, reduced population size, and marked admixture have shaped European Roma genomes. *Molecular Biology and Evolution* **37**, 3175–3187 (2020).

21. Skonieczna, K. *et al.* Heteroplasmic substitutions in the entire mitochondrial genomes of human colon cells detected by ultra-deep 454 sequencing. *Forensic Sci Int Genet* **15**, 16–20 (2015).

22. Malyarchuk, B. *et al.* Mitogenomic diversity in Russians and Poles. *Forensic Science International: Genetics* **30**, 51–56 (2017).

23. Skonieczna, K. *et al.* Mitogenomic differences between the normal and tumor cells of colorectal cancer patients. *Human Mutation* **39**, 691–701 (2018).

24. Piotrowska-Nowak, A. *et al.* Investigation of whole mitochondrial genome variation in normal tension glaucoma. *Experimental Eye Research* **178**, 186–197 (2019).

25. Piotrowska-Nowak, A. *et al.* New mtDNA association model, MutPred variant load, suggests individuals with multiple mildly deleterious mtDNA variants are more likely to suffer from Atherosclerosis. *Front. Genet.* **9**, (2019).

26. Piotrowska-Nowak, A. *et al.* Mitochondrial genome variation in Polish elite athletes. *International Journal of Molecular Sciences* **24**, 12992 (2023).

27. Gasparre, G. *et al.* Disruptive mitochondrial DNA mutations in complex I subunits are markers of oncocytic phenotype in thyroid tumors. *Proc. Nat. Acad. Sci.* **104**, 9001–9006 (2007).

28. Fernandes, V. *et al.* Genetic stratigraphy of key demographic events in Arabia. *PLoS One* **10**, e0118625 (2015).

29. Pereira, J. B. *et al.* Reconciling evidence from ancient and contemporary genomes: a major source for the European Neolithic within Mediterranean Europe. *Proc Biol Sci* **284**, 20161976 (2017).

30. Diallo, M. Y. *et al.* Circum-Saharan prehistory through the lens of mtDNA diversity. *Genes* **13**, 533 (2022).

31. Ingman, M., Kaessmann, H., Pääbo, S. & Gyllensten, U. Mitochondrial genome variation and the origin of modern humans. *Nature* **408**, 708–713 (2000).

32. Mishmar, D. *et al.* Natural selection shaped regional mtDNA variation in humans. *Proceedings of the National Academy of Sciences* **100**, 171–176 (2003).

33. Soini, H. K., Moilanen, J. S., Finnila, S. & Majamaa, K. Mitochondrial DNA sequence variation in Finnish patients with matrilineal diabetes mellitus. *BMC Res. Notes* **5**, 350 (2012).

34. Raule, N. *et al.* The co-occurrence of mtDNA mutations on different oxidative phosphorylation subunits, not detected by haplogroup analysis, affects human longevity and is population specific. *Aging Cell* **13**, 401–407 (2014).

35. Soini, H. K., Moilanen, J. S., Vilmi-Kerälä, T., Finnilä, S. & Majamaa, K. Mitochondrial DNA variant m.15218A > G in Finnish epilepsy patients who have maternal relatives with epilepsy, sensorineural hearing impairment or diabetes mellitus. *BMC Med. Genet.* **14**, 73 (2013).

36. Järviaho, T. *et al.* Novel non-neutral mitochondrial DNA mutations found in childhood acute lymphoblastic leukemia. *Clin. Genet.* **93**, 275–285 (2018).

37. Kiiskilä, J., Moilanen, J. S., Kytövuori, L., Niemi, A.-K. & Majamaa, K. Analysis of functional variants in mitochondrial DNA of Finnish athletes. *BMC Genomics* **20**, 784 (2019).

38. Lowy-Gallego, E. *et al.* Variant calling on the GRCh38 assembly with the data from phase three of the 1000 Genomes Project. *Wellcome Open Res* **4**, 50 (2019).

39. Bergström, A. *et al.* Insights into human genetic variation and population history from 929 diverse genomes. *Science* **367**, eaay5012 (2020).

40. Zaidieh, T., Smith, J. R., Ball, K. E. & An, Q. Mitochondrial DNA abnormalities provide mechanistic insight and predict reactive oxygen species-stimulating drug efficacy. *BMC Cancer* **21**, 427 (2021).

41. Dulias, K. *et al.* Ancient DNA at the edge of the world: continental immigration and the persistence of Neolithic male lineages in Bronze Age Orkney. *Proc. Nat. Acad. Sci.* **119**, e2108001119 (2022).

42. Schönberg, A., Theunert, C., Li, M., Stoneking, M. & Nasidze, I. High-throughput sequencing of complete human mtDNA genomes from the Caucasus and West Asia: high diversity and demographic inferences. *Eur. J. Hum. Genet.* **19**, 988–994 (2011).

43. Derenko, M. *et al.* Complete mitochondrial DNA diversity in Iranians. *PLoS One* **8**, e80673 (2013).

44. Derenko, M. *et al.* Insights into matrilineal genetic structure, differentiation and ancestry of Armenians based on complete mitogenome data. *Mol. Genet. Genomics* **294**, 1547–1559 (2019).

45. Carelli, V. *et al.* Haplogroup effects and recombination of mitochondrial DNA: novel clues from the analysis of Leber hereditary optic neuropathy pedigrees. *Am. J. Hum Genet.* **78**, 564–574 (2006).

46. Cerezo, M. *et al.* Reconstructing ancient mitochondrial DNA links between Africa and Europe. *Genome Res.* **22**, 821–826 (2012).

47. Olivieri, A. *et al.* Mitogenome diversity in Sardinians: a genetic window onto an Island’s past. *Mol. Biol. Evol.* **34**, 1230–1239 (2017).

48. Modi, A. *et al.* The mitogenome portrait of Umbria in Central Italy as depicted by contemporary inhabitants and pre-Roman remains. *Sci. Rep.* **10**, 10700 (2020).

49. Bandelt, H.-J. *et al.* Low “penetrance” of phylogenetic knowledge in mitochondrial disease studies. *Biochem. Biophys. Res. Commun.* **333**, 122–130 (2005).

50. Kivisild, T., Metspalu, M., Bandelt, H.-J., Richards, M. & Villems, R. The World mtDNA Phylogeny. in *Human Mitochondrial DNA and the Evolution of Homo sapiens* (eds. Bandelt, H.-J., Macaulay, V. & Richards, M.) 149–179 (Springer, Berlin, Heidelberg, 2006). doi:10.1007/3-540-31789-9_7.

51. Morgia, C. L. *et al.* Rare mtDNA variants in Leber hereditary optic neuropathy families with recurrence of myoclonus. *Neurology* **70**, 762–770 (2008).

52. Pello, R. *et al.* Mitochondrial DNA background modulates the assembly kinetics of OXPHOS complexes in a cellular model of mitochondrial disease. *Hum. Mol. Genet.* **17**, 4001–4011 (2008).

53. Zaragoza, M. V., Brandon, M. C., Diegoli, M., Arbustini, E. & Wallace, D. C. Mitochondrial cardiomyopathies: how to identify candidate pathogenic mutations by mitochondrial DNA sequencing, MITOMASTER and phylogeny. *Eur. J. Hum. Genet.* **19**, 200–207 (2011).

54. Achilli, A. *et al.* Rare primary mitochondrial DNA mutations and probable synergistic variants in Leber’s hereditary optic neuropathy. *PLoS One* **7**, e42242 (2012).

55. Perli, E. *et al.* Isoleucyl-tRNA synthetase levels modulate the penetrance of a homoplasmic m.4277T>C mitochondrial tRNAIle mutation causing hypertrophic cardiomyopathy. *Hum. Mol. Genet.* **21**, 85–100 (2012).

56. Caporali, L. *et al.* Cybrid studies establish the causal link between the mtDNA m.3890G>A/*MT*-*ND1* mutation and optic atrophy with bilateral brainstem lesions. *BBA – Mol. Basis Dis.* **1832**, 445–452 (2013).

57. Carossa, V. *et al.* A novel in-frame 18-bp microdeletion in MT-CYB causes a multisystem disorder with prominent exercise intolerance. *Hum. Mut.* **35**, 954–958 (2014).

58. Barcaccia, G., Galla, G., Achilli, A., Olivieri, A. & Torroni, A. Uncovering the sources of DNA found on the Turin Shroud. *Sci. Rep.* **5**, 14484 (2015).

59. Caporali, L. *et al.* Peculiar combinations of individually non-pathogenic missense mitochondrial DNA variants cause low penetrance Leber’s hereditary optic neuropathy. *PLoS Genet.* **14**, e1007210 (2018).

60. Girolimetti, G. *et al.* Mitochondrial DNA analysis efficiently contributes to the identification of metastatic contralateral breast cancers. *J. Cancer Res. Clin. Oncol.* **147**, 507–516 (2021).

61. Pichler, I. *et al.* Drawing the history of the Hutterite population on a genetic landscape: inference from Y-chromosome and mtDNA genotypes. *Eur. J. Hum. Genet.* **18**, 463–470 (2010).

62. Bertolin, C. *et al.* Analysis of complete mitochondrial genomes of patients with schizophrenia and bipolar disorder. *J. Hum. Genet.* **56**, 869–872 (2011).

63. Pereira, L. *et al.* No evidence for an mtDNA role in sperm motility: data from complete sequencing of asthenozoospermic males. *Mol. Biol. Evol.* **24**, 868–874 (2007).

64. Silva, M. *et al.* Biomolecular insights into North African-related ancestry, mobility and diet in eleventh-century Al-Andalus. *Sci. Rep.* **11**, 18121 (2021).

65. Davidovic, S. *et al.* Complete mitogenome data for the Serbian population: the contribution to high-quality forensic databases. *Int. J. Legal Med.* **134**, 1581–1590 (2020).

66. Borlado, M. C. G. *et al.* Impact of the mitochondrial genetic background in complex III deficiency. *PLoS One* **5**, e12801 (2010).

67. García, O. *et al.* Using mitochondrial DNA to test the hypothesis of a European post-glacial human recolonization from the Franco-Cantabrian refuge. *Heredity* **106**, 37–45 (2011).

68. Gómez-Carballa, A. *et al.* Indian signatures in the westernmost edge of the European Romani diaspora: new insight from mitogenomes. *PLoS One* **8**, e75397 (2013).

69. Gómez-Carballa, A., Pardo-Seco, J., Martinón-Torres, F. & Salas, A. Phylogenetic and population-based approaches to mitogenome variation do not support association with male infertility. *J. Hum. Genet.* **62**, 361–371 (2017).

70. Emperador, S. *et al.* The decrease in mitochondrial DNA mutation load parallels visual recovery in a Leber hereditary optic neuropathy patient. *Front. Neurosci.* **12**, (2018).

71. López-Gallardo, E. *et al.* Food derived respiratory complex I inhibitors modify the effect of Leber hereditary optic neuropathy mutations. *FCT.* **120**, 89–97 (2018).

72. Zalloua, P. *et al.* Ancient DNA of Phoenician remains indicates discontinuity in the settlement history of Ibiza. *Sci. Rep.* **8**, 17567 (2018).

73. García-Olivares, V. *et al.* Digging into the admixture strata of current-day Canary Islanders based on mitogenomes. *iScience* **26**, 105907 (2023).

74. Barbieri, C. *et al.* Contrasting maternal and paternal histories in the linguistic context of Burkina Faso. *Mol. Biol. Evol.* **29**, 1213–1223 (2012).

75. Byrska-Bishop, M. *et al.* High-coverage whole-genome sequencing of the expanded 1000 Genomes Project cohort including 602 trios. *Cell* **185**, 3426-3440.e19 (2022).

76. Barbieri, C. *et al.* Migration and Interaction in a Contact Zone: mtDNA Variation among Bantu-Speakers in Southern Africa. *PLoS One* **9**, e99117 (2014).

77. Oliveira, S. *et al.* Matriclans shape populations: Insights from the Angolan Namib Desert into the maternal genetic history of southern Africa. *Am. J. Phys. Anthropol.* **165**, 518–535 (2018).

78. Batini, C. *et al.* Insights into the demographic history of African Pygmies from complete mitochondrial genomes. *Mol. Biol. Evol.* **28**, 1099–1110 (2011).

79. Martin, A. R. *et al.* Transcriptome sequencing from diverse human populations reveals differentiated regulatory architecture. *PLoS Genet.* **10**, e1004549 (2014).

80. Brucato, N. *et al.* The Comoros show the earliest Austronesian gene flow into the Swahili corridor. *Am. J. Hum. Genet.* **102**, 58–68 (2018).

81. Pierron, D. *et al.* Genomic landscape of human diversity across Madagascar. *Proc. Nat. Acad. Sci.* **114**, E6498–E6506 (2017).

82. van der Walt, E. M. *et al.* Characterization of mtDNA variation in a cohort of South African paediatric patients with mitochondrial disease. *Eur. J. Hum. Genet.* **20**, 650–656 (2012).

83. Pickrell, J. K. *et al.* The genetic prehistory of southern Africa. *Nat. Commun.* **3**, 1143 (2012).

84. Matisoo-Smith, E. *et al.* Ancient mitogenomes of Phoenicians from Sardinia and Lebanon: A story of settlement, integration, and female mobility. *PLoS One* **13**, e0190169 (2018).

85. Abu-Amero, K. K., Larruga, J. M., Cabrera, V. M. & González, A. M. Mitochondrial DNA structure in the Arabian Peninsula. *BMC Evol. Biol.* **8**, 45 (2008).

86. Aljasmi, F. A. *et al.* Genomic landscape of the mitochondrial genome in the United Arab Emirates native population. *Genes* **11**, 876 (2020).

87. Hippen, M. *et al.* Novel pathogenic sequence variation m.5789T>C causes NARP syndrome and promotes formation of deletions of the mitochondrial genome. *Neurol. Genet.* **8**, e660 (2022).

88. Behar, D. M. *et al.* A “Copernican” reassessment of the human mitochondrial DNA tree from its root. *Am. J. Hum. Genet.* **90**, 675–684 (2012).

89. Bodner, M. *et al.* Helena’s many daughters: more mitogenome diversity behind the most common west Eurasian mtDNA control region haplotype in an extended Italian population sample. *Int. J. Mol. Sci.* **23**, 6725 (2022).

90. Ottoni, C. *et al.* Mitochondrial haplogroup H1 in North Africa: an early Holocene arrival from Iberia. *PLoS One* **5**, e13378 (2010).

91. Pala, M. *et al.* Mitochondrial DNA signals of Late Glacial recolonization of Europe from Near Eastern Refugia. *Am. J. Hum. Genet.* **90**, 915–924 (2012).

92. Behar, D. M. *et al.* The dawn of human matrilineal diversity. *Am. J. Hum. Genet.* **82**, 1130–1140 (2008).

93. Hernández, C. L. *et al.* Early holocenic and historic mtDNA African signatures in the Iberian Peninsula: the Andalusian region as a paradigm. *PLoS One* **10**, e0139784 (2015).

94. Just, R. S., Irwin, J. A. & Parson, W. Mitochondrial DNA heteroplasmy in the emerging field of massively parallel sequencing. *Forensic Sci. Int. Genet.* **18**, 131–139 (2015).

95. Posth, C. et al. Pleistocene mitochondrial genomes suggest a single major dispersal of non-Africans and a late glacial population turnover in Europe. *Curr. Biol.* **26**, 827–833 (2016).
